# Polygenic risk scores for schizophrenia are associated with oculomotor endophenotypes

**DOI:** 10.1017/S0033291721003251

**Published:** 2023-03

**Authors:** Annabell Coors, Mohammed-Aslam Imtiaz, Meta M. Boenniger, N. Ahmad Aziz, Monique M. B. Breteler, Ulrich Ettinger

**Affiliations:** 1 Population Health Sciences, German Center for Neurodegenerative Diseases (DZNE), Bonn, Germany; 2 Department of Neurology, Faculty of Medicine, University of Bonn, Bonn, Germany; 3 Institute for Medical Biometry, Informatics and Epidemiology (IMBIE), Faculty of Medicine, University of Bonn, Bonn, Germany; 4 Department of Psychology, University of Bonn, Bonn, Germany

**Keywords:** Antisaccade, epidemiology, eye movement, genetic risk score, genetics, prosaccade, smooth pursuit

## Abstract

**Background:**

Schizophrenia is a heterogeneous disorder with substantial heritability. The use of endophenotypes may help clarify its aetiology. Measures from the smooth pursuit and antisaccade eye movement tasks have been identified as endophenotypes for schizophrenia in twin and family studies. However, the genetic basis of the overlap between schizophrenia and these oculomotor markers is largely unknown. Here, we tested whether schizophrenia polygenic risk scores (PRS) were associated with oculomotor performance in the general population.

**Methods:**

Analyses were based on the data of 2956 participants (aged 30–95) of the Rhineland Study, a community-based cohort study in Bonn, Germany. Genotyping was performed on Omni-2.5 exome arrays. Using summary statistics from a recent meta-analysis based on the two largest schizophrenia genome-wide association studies to date, we quantified genetic risk for schizophrenia by creating PRS at different *p* value thresholds for genetic markers. We examined associations between PRS and oculomotor performance using multivariable regression models.

**Results:**

Higher PRS were associated with higher antisaccade error rate and latency, and lower antisaccade amplitude gain. PRS showed inconsistent patterns of association with smooth pursuit velocity gain and were not associated with saccade rate during smooth pursuit or performance on a prosaccade control task.

**Conclusions:**

There is an overlap between genetic determinants of schizophrenia and oculomotor endophenotypes. Our findings suggest that the mechanisms that underlie schizophrenia also affect oculomotor function in the general population.

## Background

Schizophrenia is a severe mental disorder with a lifetime prevalence of just under 1% (McGrath, Saha, Chant, & Welham, 2008). There is substantial evidence for a genetic basis of schizophrenia, with recurrence risk in families of about 8.6% (Lichtenstein et al., 2006) and heritability estimates of up to 81% (Sullivan, Kendler, & Neale, 2003). Genome-wide association studies (GWASs) thus far have identified 145 single nucleotide polymorphisms (SNPs) that are associated with schizophrenia (Pardiñas et al., 2018). However, the genetic variance of schizophrenia explained by these SNPs is low (Pardiñas et al., 2018). On the one hand, there are many SNPs that do not reach the genome-wide significant threshold in a GWAS (5 × 10^−8^) but that could explain in sum a substantial proportion of genetic variance (International Schizophrenia Consortium, 2009). Another reason for this may be that schizophrenia is highly heterogeneous and encompasses a multitude of different syndromes that do not necessarily have a common biological basis (Braff, Freedman, Schork, & Gottesman, 2007).

Endophenotypes have been proposed as an approach to better understand the aetiology of schizophrenia (Braff et al., 2007). They are considered to link a disorder to its genetic basis and to be closer to the actions of genes than disease symptoms are (Gottesman & Gould, 2003). Furthermore, as current disease classification might not well reflect aetiology (The Brainstorm Consortium, 2018), endophenotypes may help identify more homogenous subgroups of patients with shared biological basis (Braff et al., 2007).

Deficits in antisaccade and smooth pursuit eye movement (SPEM) tasks are amongst the best replicated endophenotypes for schizophrenia (Calkins, Iacono, & Ones, 2008; Holzman, 2000).

In the antisaccade task, participants are required to make a saccade in the opposite direction to a sudden-onset, peripheral target (Hallett, 1978). Individuals with schizophrenia make more antisaccade errors (trials in which the initial saccade is erroneously executed towards the peripheral target) compared to controls (Clementz, McDowell, & Zisook, 1994; Ettinger et al., 2004; Fukushima et al., 1988; Radant et al., 2010; Reilly et al., 2014; Reuter, Rakusan, & Kathmanna, 2005; Sereno & Holzman, 1995). Higher antisaccade latencies (time needed to initiate the first saccade after the appearance of the peripheral target) have also been reported in some (Curtis, Calkins, Grove, Feil, & Iacono, 2001; Ettinger et al., 2004; Fukushima et al., 1988; Fukushima et al., 1990b; Fukushima, Fukushima, Morita, & Yamashita, 1990a; Karoumi et al., 2001; Mazhari et al., 2011; Sereno & Holzman, 1995) but not all studies (Radant et al., 2007, 2010; Reuter et al., 2005). Antisaccade amplitude gain (a measure of spatial accuracy of directionally correct antisaccades) was found to be reduced in individuals with schizophrenia in some (Ettinger et al., 2004; Karoumi et al., 2001; Radant et al., 2010) but not all studies (Ettinger et al., 2018; Fukushima et al., 1990a). However, impaired antisaccade performance has been reported in patients with psychosis across different diagnostic categories (Reilly et al., 2014) and, therefore, may have relatively low specificity for schizophrenia.

In the SPEM task, participants follow a slowly moving target with their eyes. Patients with schizophrenia have long been known to have SPEM impairments (Diefendorf & Dodge, 1908; Holzman, Proctor, & Hughes, 1973), characterised primarily by lower velocity gain (ratio of eye and target velocity) and higher saccade rates than controls (O'Driscoll & Callahan, 2008; Sereno & Holzman, 1995). Greater deficits in antisaccade error rate have also been observed in patients with schizophrenia who have impaired SPEM performance compared to patients with schizophrenia without SPEM impairments (Sereno & Holzman, 1995).

Antisaccade and pursuit measures have moderate-to-high heritability (Bell, Abel, Li, Christian, & Yee, 1994; Greenwood et al., 2007; Hong et al., 2006; Katsanis, Taylor, Iacono, & Hammer, 2000; Litman et al., 1997; Macare, Meindl, Nenadic, Rujescu, & Ettinger, 2014; Malone & Iacono, 2002) and temporal stability (Calkins, Iacono, & Curtis, 2003; Campion et al., 1992; Crevits, De Clerck, & Van Maele, 2000; Ettinger et al., 2003; Flechtner, Steinacher, Sauer, & Mackert, 2002; Gooding, Mohapatra, & Shea, 2004; Light et al., 2012; Sweeney et al., 1999). Clinically unaffected first-degree relatives of schizophrenia patients show impairments similar to those seen in patients with schizophrenia, albeit with smaller effect sizes (Calkins et al., 2008).

In contrast, performance in prosaccade tasks is typically preserved in schizophrenia (Damilou, Apostolakis, Thrapsanioti, Theleritis, & Smyrnis, 2016; Ettinger et al., 2018; Fukushima et al., 1988; Fukushima et al., 1990a; Fukushima et al., 1990b), although some studies have observed reduced spatial accuracy (Schmid-Burgk, 1984; Schreiber et al., 1995).

Overall, these findings suggest an overlap in the genetic determinants of specific oculomotor endophenotypes and schizophrenia. However, schizophrenia candidate gene studies have revealed only limited and inconsistent associations with oculomotor endophenotypes (Gatt, Burton, Williams, & Schofield, 2015; Greenwood, Light, Swerdlow, Radant, & Braff, 2012; Haraldsson et al., 2009, 2010; Kattoulas et al., 2012; Rybakowski, Borkowska, Czerski, & Hauser, 2002; Thaker, Wonodi, Avila, Hong, & Stine, 2004). Thus, despite evidence of genetic overlap between eye movements and schizophrenia from family studies (Calkins et al., 2003, 2008; Levy, Sereno, Gooding, & O'Driscoll, 2010), evidence from molecular genetic studies is largely missing.

Here, we used SNPs previously associated with schizophrenia in GWASs to investigate whether genetic determinants of schizophrenia are associated with oculomotor endophenotypes in a large population-based cohort study. To this end, we calculated polygenic risk scores (PRS) based on the summary statistics of the largest schizophrenia GWAS to date (Pardiñas et al., 2018). The SNPs that have been identified so far in the schizophrenia GWAS have been associated with, inter alia, voltage-gated calcium channels, synaptic transmission, membrane depolarisation during action potentials, and fragile X mental retardation protein (FMRP) (Pardiñas et al., 2018). We hypothesised that higher PRS would be associated with worse antisaccade and SPEM performance (higher error rate, latency and saccade frequency during SPEM but lower amplitude gain and SPEM velocity gain) but unrelated to performance in the prosaccade control task (latency and amplitude gain).

## Material and methods

### Participants

We used data from participants of the Rhineland Study, a community-based cohort study in Bonn, Germany. All inhabitants of two geographically defined areas in Bonn who are 30 years or older are invited to participate in the Rhineland Study. Names and addresses were provided by the municipality. Study participation is possible upon invitation only and irrespective of health status. The only exclusion criterion is not having sufficient command of the German language to provide written informed consent. There are no financial incentives for study participation. The ethics committee of the Medical Faculty of the University of Bonn approved the study, which was carried out in accordance with the recommendations of the International Council for Harmonisation Good Clinical Practice standards (ICH-GCP). We restricted our sample to the first 4000 participants of the Rhineland Study. Since study recruitment is ongoing, we cannot provide information on general response rates, but 3523 participants (88.1%) of those first 4000 participants provided blood samples between March 2016 and July 2019. Of those, 3217 (91.3%) remained after quality control of genetic data (see section ‘Genetic data and polygenic risk scores’). Of those, 250 participants (7.8%) had no SPEM and antisaccade data. Missing data were primarily due to technical issues during data acquisition and post-processing (74%), exclusion after visual inspection of data (9.6%), contraindications (8.8%), non-compliance (5.2%), refusal (0.8%), timeout (0.4%) or multiple of these reasons (1.2%). Finally, we excluded two individuals with a diagnosis of schizophrenia and nine individuals with a diagnosis of psychosis. Thus, we based our analysis on 2956 participants without schizophrenia or psychosis aged between 30 and 95 years.

### Genetic data and polygenic risk scores

Genotyping of 3523 blood samples was performed using Illumina's Omni-2.5 exome arrays containing 2 612 357 SNPs. We processed genotype data with GenomeStudio (version 2.0.5), and performed quality control of the genotypes with PLINK (version 1.9) (Purcell et al., 2007). SNPs were excluded based on Hardy–Weinberg disequilibrium (*p* < 1 × 10^−6^), minor allele frequencies (<0.01) and poor genotyping rate (<99%) (Marees et al., 2018). Further, we removed participants with poor DNA samples as identified by poor call rate (<95%) (*N* = 8, 0.2%), abnormal heterozygosity (*N* = 47, 1.3%), cryptic relatedness (*N* = 143, 4.1%) and gender mismatch (*N* = 7, 0.2%). We used EIGENSTRAT (version 16000), which uses principal components to detect and correct for variation in population structure as this can cause systematic differences in allele frequencies (Price et al., 2006) [exclusion of *N* = 101 (2.9%) non-Caucasian participants]. Finally, missing SNPs were imputed using IMPUTE version 2 software (Howie, Donnelly, & Marchini, 2009) based on the 1000 Genomes reference panel (Auton et al., 2015). Imputation quality of the SNPs was checked using the info score metric [values of >0.3 are considered to indicate reliable imputation quality (Verma et al., 2014)].

PRS for schizophrenia were created using summary statistics from the largest schizophrenia GWAS to date, which included a discovery sample of 40 675 schizophrenia cases and 64 643 controls and an independent replication sample of 5762 cases and 154 224 controls (Pardiñas et al., 2018; Ripke et al., 2014). The results are publicly available (https://walters.psycm.cf.ac.uk; last retrieved at: 2021/05/31). We calculated PRS using PLINK (version 1.9) (Purcell et al., 2007) by first multiplying the number of risk alleles by the known effect size of each individual SNP locus and then aggregating the weighted effects of all SNPs under consideration (International Schizophrenia Consortium, 2009). We first created PRS based on the pre-specified SNPs from the GWAS, i.e. the 145 SNPs that reached genome-wide significance in the GWAS (Pardiñas et al., 2018). Then, we applied clumping to identify the most significant SNPs per linkage disequilibrium (LD) block (kilo base pair window: 250, LD *r*^2^ < 0.1) (Chasioti, Yan, Nho, & Saykin, 2019) and created PRS at *p* value threshold (*p*_T_) for SNP inclusion of 0.01 and 0.05, since PRS at those thresholds were reported to have improved prediction accuracy (Jonas et al., 2019; Ripke et al., 2014; Toulopoulou et al., 2019; Zhang et al., 2019). For sensitivity analysis, we created two additional PRS. We created one PRS by first applying clumping and then using the genome-wide significant threshold for SNP inclusion (*p*_T_ = 5 × 10^−8^), and another PRS using a more lenient threshold (*p*_T_ = 0.1), as in previous studies (Toulopoulou et al., 2019; Zhang et al., 2019).

### Eye movement data

A detailed description of oculomotor data acquisition and processing has been published (Coors et al., 2021). In brief, eye movements were recorded using video-based infrared oculography (EyeLink 1000 and EyeLink 1000 Plus; SR Research Ltd, Ottawa, Canada) at 1000 Hz. After a horizontal-vertical five-point calibration task, participants performed fixation (not reported here), SPEM, prosaccade and antisaccade tasks in fixed order. SPEM outcomes were velocity gain (in %) and saccade rate (given in *N*/s, across the entire task duration). Prosaccade outcomes were latency (in ms) and amplitude gain (saccade amplitude divided by target step amplitude). Antisaccade outcomes were error rate (in %), latency and amplitude gain. Prosaccade and antisaccade outcomes were only calculated if there were at least seven valid trials. In case of more than four antisaccade errors, there also had to be at least one corrective saccade to ensure that participants understood the instructions. Additionally, latency and amplitude gain were only calculated if there were at least seven valid trials with directionally correct initial saccades. Before applying those criteria, we performed sensitivity analysis to rule out the possibility that they led to the exclusion of the participants with the highest PRS as this could have explained invalid or poor performance. Since we found no systematic pattern, we excluded those cases (51 participants for antisaccade error rate, 305 participants for antisaccade latency and amplitude gain and 14 participants for prosaccade latency and amplitude gain).

### Statistical analyses

We hypothesised that high genetic risk for schizophrenia would be associated with worse antisaccade and SPEM performance but not with prosaccade outcomes (Calkins et al., 2008).

First, linear regression model assumptions were tested with diagnostic plots (scale-location plot and quantile-quantile plot) and by calculating the variance inflation factor [R package car (Fox & Weisberg, 2019), vif-function]. For prosaccade and antisaccade latency, the normality assumption was violated and therefore we log-transformed those variables.

Then, we assessed the associations between PRS and oculomotor outcomes with separate multivariable linear regression models for SPEM, antisaccade and prosaccade outcomes. Regression models included *z*-standardised PRS as a predictor and were adjusted for age, age^2^, sex and population stratification. For the latter, we calculated six principal components that we included as covariates in the model (Price et al., 2006). We used mean-centred age to reduce collinearity (Iacobucci, Schneider, Popovich, & Bakamitsos, 2016). Missing covariate data were imputed using predictive mean matching [Hmisc package, 10 bootstrap replicates (Harrell & Dupont, 2020)].

We did not correct for multiple testing as we had very specific *a priori* hypotheses regarding associations between the schizophrenia PRS and eye movement outcomes based on work that goes back decades (Diefendorf & Dodge, 1908; Fukushima et al., 1988; Holzman et al., 1973; Sereno & Holzman, 1995). Further, we included the prosaccade task as control condition and created additional PRS for sensitivity analysis. As argued elsewhere, correction for multiple testing is strongly context-dependent and can lead to misinterpretation of results if incorrectly applied (Rothman, 1990; Streiner & Norman, 2011). Multiple testing is considered inappropriate for a limited set of pre-specified hypotheses and becomes especially problematic if the statistical tests are not independent, which is clearly the case in our analyses where our predictors (i.e. PRS scores at different *p* value thresholds) are highly correlated (Streiner & Norman, 2011).

Given the large age range of our sample, we additionally tested whether the associations between PRS and eye movement outcomes varied with age using a likelihood ratio test. We also repeated the analyses in an age-truncated sample (participants aged 30–70 years).

Statistical analyses were performed in RStudio (version 1.1.447, R-base version 3.5.0), using an *α* level of 0.05.

## Results

### Study sample

Sample characteristics are presented in Table 1.

**Table 1.** Sample characteristics

Number of participants, *N* (%)	2956 (100)
30–39 years	506 (17.1)
40–49 years	559 (18.9)
50–59 years	782 (26.5)
60–69 years	565 (19.1)
70–79 years	421 (14.2)
80+ years	123 (4.2)
Age, *M* (s.d.) in years	55.1 (14.2)
Sex, *N* (%) women	1665 (56.3)
Education level, *N* (%)	2935 (99.3)
High	1578 (53.8)
Middle	1309 (44.6)
Low	48 (1.6)
Best-corrected visual acuity, *N* (%)	2956 (100)
High (⩾0.8)	2536 (85.8)
Middle (0.32–0.63)	381 (12.9)
Low (<0.32)	39 (1.3)
Antisaccade error rate [%], *M* (s.d.)	32.1 (24.1)
Antisaccade latency [ms], median (interquartile range)	273.9 (59.2)
Antisaccade amplitude gain [%], *M* (s.d.)	111.6 (27.9)
Smooth pursuit velocity gain [%], *M* (s.d.)	77.8 (16.7)
Saccade frequency during smooth pursuit [*N*/s], *M* (s.d.)	2.2 (0.6)
Prosaccade latency [ms], median (interquartile range)	186.4 (35.8)
Prosaccade amplitude gain [%], *M* (s.d.)	93.3 (6.9)

*N* = number of participants, *M* = mean, s.d. = standard deviation. Education level was determined using the International Standard Classification of Education 2011 (ISCED) and was coded as low (lower secondary education or below), middle (upper secondary education to undergraduate university level) and high (postgraduate university study). Assessment of best-corrected visual acuity was based on visual scores from the right eye and was measured using an automated refractometer (Ark-1s, NIDEK CO., Tokyo, Japan). Categorisation of the visual acuity values was based on the guidelines of the International Council of Ophthalmology.

### Associations between PRS for schizophrenia and oculomotor performance

The main results from the multivariable regression models are listed in Table 2 and the results of the PRS that we created for sensitivity analysis are in online Supplementary Table A.1. PRS was positively associated with antisaccade error rate irrespective of inclusion criteria (pre-specified SNPs, *p*_T_ = 0.01, *p*_T_ = 0.05), but sensitivity analysis at *p*_T_ = 5 × 10^−8^ and *p*_T_ = 0.1 was not significant. For antisaccade latency and amplitude gain, PRS at *p*_T_ = 0.01 and *p*_T_ = 0.05 were associated with those outcomes but PRS including the pre-specified SNPs and PRS created for sensitivity analysis were not. The associations were positive for antisaccade latency and negative for antisaccade amplitude gain. PRS at *p*_T_ = 0.01 was positively associated with SPEM velocity gain but the other two PRS (pre-specified SNPs, *p*_T_ = 0.05) were not significantly associated with it. In addition, sensitivity analysis revealed a positive association between PRS at *p*_T_ = 0.1 and SPEM velocity gain. None of the PRS was associated with saccade rate. Regarding the prosaccade control task, none of the PRS was significantly associated with latency or amplitude gain.

**Table 2.** Associations between polygenic risk scores (PRS) for schizophrenia at different *p* value thresholds for SNP inclusion and eye movement outcomes

Eye movement outcome	*p* value threshold for SNP inclusion	*b* (95% CI) for PRS	*p* value	*R*^2^ (%)
Antisaccade error rate (%)	Pre-specified SNPs	**0.897 (0.058 to 1.737)**	**0.036**	**0.1**
Antisaccade error rate (%)	0.01	**1.007 (0.056 to 1.958)**	**0.038**	**0.1**
Antisaccade error rate (%)	0.05	**1.104 (0.169 to 2.039)**	**0.021**	**0.1**
Log of antisaccade latency (log ms)	Pre-specified SNPs	0.002 (0.000 to 0.005)	0.092	–
Log of antisaccade latency (log ms)	0.01	**0.004 (0.001 to 0.007)**	**0.012**	**0.2**
Log of antisaccade latency (log ms)	0.05	**0.003 (0.000 to 0.006)**	**0.039**	**0.1**
Antisaccade amplitude gain (%)	Pre-specified SNPs	−0.193 (−1.295 to 0.909)	0.731	**–**
Antisaccade amplitude gain (%)	0.01	**−1.489 (−2.723 to −0.254)**	**0.018**	**0.2**
Antisaccade amplitude gain (%)	0.05	**−1.279 (−2.491 to −0.067)**	**0.039**	**0.1**
Smooth pursuit velocity gain (%)	Pre-specified SNPs	−0.005 (−0.536 to 0.525)	0.984	–
Smooth pursuit velocity gain (%)	0.01	**0.647 (0.048 to 1.247)**	**0.034**	**0.1**
Smooth pursuit velocity gain (%)	0.05	0.476 (−0.113 to 1.066)	0.113	–
Saccade frequency during smooth pursuit (*N*/s)	Pre-specified SNPs	−0.005 (−0.025 to 0.015)	0.632	–
Saccade frequency during smooth pursuit (*N*/s)	0.01	−0.013 (−0.035 to 0.010)	0.262	–
Saccade frequency during smooth pursuit (*N*/s)	0.05	0.001 (−0.021 to 0.023)	0.941	–
Prosaccade amplitude gain (%)	Pre-specified SNPs	−0.153 (−0.397 to 0.091)	0.218	–
Prosaccade amplitude gain (%)	0.01	−0.122 (−0.399 to 0.154)	0.385	–
Prosaccade amplitude gain (%)	0.05	−0.033 (−0.304 to 0.238)	0.813	–
Log of prosaccade latency (log ms)	Pre-specified SNPs	0.000 (−0.002 to 0.002)	0.937	–
Log of prosaccade latency (log ms)	0.01	−0.002 (−0.004 to 0.000)	0.112	–
Log of prosaccade latency (log ms)	0.05	−0.002 (−0.004 to 0.001)	0.153	–

The table displays the change in performance per one standard deviation increase in PRS for schizophrenia for different eye movement outcomes. *b*, unstandardised regression coefficient; 95% CI, 95% confidence interval. Unstandardised regression coefficients were obtained from the following multivariable linear regression model: Eye movement outcome ~*b*_0_ + PRS × *b*_1_ + age + age^2^ + sex + population stratification + residual error. *R*^2^ refers to the variance explained in eye movement performance by PRS in per cent. In bold are those associations with a *p* value below 0.05.

Effects for all associations were small, with at most 0.22% of variance in oculomotor outcomes explained by the PRS.

We found no interaction effects between age and PRS. Effect estimates in the age-truncated analysis were highly comparable to those in the whole sample, but given the smaller sample size (*N* = 2636), some confidence intervals were wider. For the association between PRS at *p*_T_ = 0.05 and antisaccade amplitude gain, this resulted in the inclusion of zero in the confidence interval, but the regression coefficient remained comparable (full sample: *b* = −1.279; 95% CI −2.491 to −0.067; age-truncated sample: *b* = −1.228; 95% CI −2.520 to 0.064).

## Discussion

We investigated genetic determinants of schizophrenia in relation to oculomotor endophenotypes in a large, population-based cohort. We found that genetic variants that are associated with schizophrenia are also involved in the fine-regulation of particular aspects of oculomotor function. Schizophrenia-related genetic risk variants specifically affected antisaccade outcomes, but not saccade rate during SPEM or outcomes from the prosaccade control task. PRS showed inconsistent patterns of association with SPEM velocity gain. Whilst collectively these findings thus support the use of specific oculomotor endophenotypes as markers of those syndromes that are currently classified as schizophrenia, it should be noted that the effect sizes of the observed associations are small.

Our findings suggest that SNP inclusion thresholds of *p*_T_ = 0.01 and *p*_T_ = 0.05 were optimal for the detection of PRS correlates of eye movements, in line with the findings of previous studies (Jonas et al., 2019; Ripke et al., 2014; Toulopoulou et al., 2019; Zhang et al., 2019). Of the PRS that we calculated for sensitivity analysis, the *p*_T_ = 0.1 cut-off might have been less optimal for this study as a more lenient threshold implies the inclusion of more uninformative SNPs and, therefore, an increase in noise (Chasioti et al., 2019). On the contrary, PRS at the genome-wide significant *p*_T_ (*p*_T_ = 5 × 10^−8^) might have excluded too many informative SNPs (Pardiñas et al., 2018). Our sensitivity analysis showed that applying clumping and then applying the genome-wide significant threshold obscured the association between PRS and antisaccade error rate that we found when we used PRS based on only the pre-specified SNPs.

Supporting the endophenotype status of antisaccade latency and error rate, we found both to be positively associated with genetic risk for schizophrenia which is in line with the reports of deficits in these measures in patients with schizophrenia and their clinically unaffected relatives (Calkins et al., 2008; Curtis et al., 2001; Ettinger et al., 2004; Fukushima et al., 1988; Fukushima et al., 1990a; Fukushima et al., 1990b; Karoumi et al., 2001; Mazhari et al., 2011; Radant et al., 2007, 2010; Reilly et al., 2014; Reuter et al., 2005; Sereno & Holzman, 1995). Antisaccade latency depends on cognitive processes such as attention, response-related decision-making and response execution (Hutton, 2008) and has been linked to activity in saccade neurons in the frontal eye fields and superior colliculus (Munoz & Everling, 2004). Reduced activation of frontal eye fields in individuals with schizophrenia compared to controls during eye movement tasks has been reported (Keedy, Ebens, Keshavan, & Sweeney, 2006) and may, therefore, partly account for the association between PRS and latency. Successful inhibition of antisaccade errors has been associated with activity in the dorsolateral prefrontal cortex (Munoz & Everling, 2004). Since individuals with schizophrenia have been found to have lower activity in prefrontal areas during antisaccade task performance than controls (McDowell et al., 2002), the association between genetic risk for schizophrenia and antisaccade error rate may be partly mediated by prefrontal areas, although the striatum may also play a role (Raemaekers, Ramsey, Vink, van den Heuvel, & Kahn, 2006, 2002).

The negative association between genetic risk for schizophrenia and antisaccade amplitude gain is also in line with studies reporting lower antisaccade amplitude gain in schizophrenia patients (Ettinger et al., 2004; Karoumi et al., 2001; Radant et al., 2010) and their biological relatives (Ettinger et al., 2018, 2006, 2004; Karoumi et al., 2001), again supporting the endophenotype candidacy of this measure. However, in those studies, the mean antisaccade accuracy of controls typically ranged between 95% and 100% and, therefore, lower amplitude gain in patients or relatives indicated lower spatial accuracy. Instead, participants in our study on average tended to make hypermetric (overshooting) antisaccades (*M* = 111.7%, s.d. = 29.1; Table 1), implying that participants with higher PRS had in fact greater absolute spatial accuracy, as their scores were closer to 100%. Antisaccade amplitude gain values above 100% are not unusual and values comparable to ours have been reported by others (Sweeney, Rosanao, Berman, & Luna, 2001). Antisaccade spatial accuracy requires complex, non-standard sensorimotor transformations in the posterior parietal cortex (Herweg et al., 2014) and frontal eye fields (Moon et al., 2007). Together, these findings confirm a genetic overlap between schizophrenia and antisaccade amplitude gain and point to a general tendency of people with a higher genetic risk of schizophrenia to make antisaccades with lower amplitudes.

However, since schizophrenia is a highly heterogeneous disease (Braff et al., 2007) and antisaccade performance has rather a low specificity for schizophrenia (Reilly et al., 2014), we cannot exclude the possibility that the associations between PRS for schizophrenia and antisaccade outcomes may also be partly accounted for by genetic risk variants of other psychiatric disorders.

The finding of *higher* SPEM velocity gain and, therefore, better performance in participants with higher PRS at *p*_T_ = 0.01 was unexpected, given the highly consistent reports of *lower* velocity gain in patients with schizophrenia and their relatives compared to healthy controls (Calkins et al., 2008; O'Driscoll & Callahan, 2008). An explanation might be that higher genetic risk for schizophrenia but not having schizophrenia may be advantageous for performance in SPEM velocity gain. Genes associated with schizophrenia were found to be favoured by evolution which implies that they might be advantageous for (cognitive) functioning, at least to a certain degree (Banerjee et al., 2018; Srinivasan et al., 2016). However, it is unclear why those advantages should only exist in performance in one but not other oculomotor endophenotypes. In contrast to antisaccade outcomes, SPEM velocity gain was associated with PRS at *p*_T_ = 0.01 and *p*_T_ = 0.1 but unrelated to PRS at *p*_T_ = 0.05. Since this is the only eye movement outcome for which the pattern was inconsistent for PRS at *p*_T_ = 0.01 and *p*_T_ = 0.05, and since the inclusion of more than the genome-wide significant SNPs in PRS creation may also increase the level of noise (Chasioti et al., 2019), an alternative explanation is that the relation of higher PRS with higher SPEM velocity gain may have been a false-positive finding.

Despite previous findings of higher saccade rate during SPEM in schizophrenia patients (O'Driscoll & Callahan, 2008) and their relatives (Calkins et al., 2008), we did not find an association of PRS with that measure. This could be due to either a limited overlap in the genetic determinants between schizophrenia and saccade rate, or the current PRS capturing only a small proportion of those shared genetic factors.

Our sensitivity analysis of the prosaccade control task showed that PRS were not associated with prosaccade latency and amplitude gain. Since performance in these outcomes is largely unaffected in patients with schizophrenia and their relatives (Calkins et al., 2008; Reilly et al., 2014), this finding corroborates our explicit *a priori* hypothesis that associations between PRS for schizophrenia and oculomotor outcomes are limited to those oculomotor outcomes that have been established as endophenotypes of schizophrenia.

From a genetic perspective, it is noteworthy that those SNPs that we included in the PRS were found to be associated with, *inter alia*, voltage-gated calcium channels and the FMRP (Pardiñas et al., 2018). Voltage-gated calcium channels play a key role in visual perception (Pangrsic, Singer, & Koschak, 2018) and mutations have been linked to visual deficits such as involuntary eye movements (Cain & Snutch, 2011). In mice, FMRP has been associated with prefrontal cortex dysfunction (Siegel et al., 2017), which is also a critical brain structure for successful antisaccade performance (Kaufman, Pratt, Levine, & Black, 2010). This suggests that shared mechanisms may underlie schizophrenia and oculomotor performance and that those shared mechanisms may become particularly evident in some specific oculomotor outcomes. However, we also know from genetics that there are systematic differences in allele frequencies between populations (Price et al., 2006). Thus, our findings may not be generalisable to non-Caucasian populations as we based our PRS on SNPs derived from a study including predominantly Caucasians (Wand et al., 2021).

Our sample included individuals aged between 30 and 95 years. Previous studies found that the majority of patients with schizophrenia develop the disease during adolescence and early adulthood (men: between age 10 and 25, women: between age 25 and 35) (Rajji, Ismail, & Mulsant, 2009). Approximately one-quarter of patients with schizophrenia, and particularly women, experience their first episode after the age of 40 and very few patients are diagnosed after age 60 (Rajji et al., 2009). Thus, the probability that our population-based sample included individuals that are about to develop schizophrenia but have not yet been diagnosed is very low. Our large sample size and the young typical age of onset benefit our research aim as these factors lower the risk that the observed associations between schizophrenia PRS and eye movement performance were due to individuals about to develop schizophrenia. We found no evidence that the observed associations varied with age, yet we lacked the statistical power to run age group-specific analyses in more narrow age ranges. Further research, conducted in large samples with narrower age ranges, is needed to confirm the associations we found between genetic liability for schizophrenia and oculomotor measures.

The observation that only small amounts of variance in established oculomotor endophenotypes could be explained by PRS needs critical examination. One possibility is that current PRS do not fully capture the genetic basis of schizophrenia. The estimate for the common-variant SNP heritability of schizophrenia calculated in the largest GWAS to date is 24.4% if all SNPs are considered, and only 6% for PRS at *p*_T_ = 0.05 (Pardiñas et al., 2018). These estimates are, therefore, well below family-based studies heritability estimates (Lichtenstein et al., 2009; Sullivan et al., 2003). Current PRS may capture only a fraction of genetic variance attributed to schizophrenia because even the largest GWASs to date were not sufficiently powered to detect all relevant common variants (Smeland, Frei, Dale, & Andreassen, 2020). In addition, part of the heritability results from copy number variants or rare variants that influence the boundaries of topologically associated domains (Halvorsen et al., 2020; Marshall et al., 2017), which are currently not tagged by conventional genotyping arrays and, therefore, not included in GWAS (Auer & Lettre, 2015).

Further, it should be remembered that heritability estimates for both schizophrenia (Lichtenstein et al., 2009; Sullivan et al., 2003) and eye movements (Bell et al., 1994; Greenwood et al., 2007; Hong et al., 2006; Katsanis et al., 2000; Litman et al., 1997; Macare et al., 2014; Malone & Iacono, 2002) are well below 100%. This, and the only modest sized oculomotor impairments in first-degree relatives of schizophrenia patients (Calkins et al., 2008), implies that oculomotor impairments in schizophrenia reflect not only genetic but also environmental factors as well as the interplay between genes and environment (Chakravarti & Little, 2003).

It should also be noted that our inclusion criterion of a minimum age of 30 years, combined with the typically rather early onset for schizophrenia (Rajji et al., 2009), may have led to the exclusion of some participants with very high genetic risk for schizophrenia, thereby reducing the variance in schizophrenia risk and oculomotor performance in our sample.

Taken together, the schizophrenia PRS alone is unlikely to fully account for differences in oculomotor performance. This also fits with our finding that current schizophrenia PRS do not have any predictive power for eye movement performance. Still, the molecular genetic confirmation implies that the role of those brain regions that are critically involved in antisaccade performance should be investigated more closely in the aetiology of schizophrenia. Thus, combining knowledge from eye movement and schizophrenia research could be beneficial to propel the field forward.

## Conclusions

Using a molecular genetic approach, we confirm and extend previous findings from behavioural genetic studies, showing that antisaccade error rate, latency and amplitude gain have genetic overlap with schizophrenia. For SPEM outcomes, we found no association between PRS and saccade rate and inconsistent associations between PRS and velocity gain. As schizophrenia PRS based on currently available GWAS findings only accounted for <0.25% of variance in oculomotor endophenotypes, they currently have no predictive power. However, we expect that future studies using PRS that also include rare risk variants are likely to uncover a larger proportion of shared genetic determinants of schizophrenia and oculomotor performance.

## Supplementary material

For supplementary material accompanying this paper visit https://doi.org/10.1017/S0033291721003251. click here to view supplementary material

## References

[ref3] Auer, P. L., & Lettre, G. (2015). Rare variant association studies: Considerations, challenges and opportunities. Genome Medicine, 7, 16. 10.1186/s13073-015-0138-2. 25709717 PMC4337325

[ref4] Auton, A., Abecasis, G. R., Altshuler, D. M., Durbin, R. M., Bentley, D. R., Chakravarti, A., … Schloss, J. A. (2015). A global reference for human genetic variation. Nature, 526, 68–74. 10.1038/nature15393. 26432245 PMC4750478

[ref5] Banerjee, N., Polushina, T., Bettella, F., Giddaluru, S., Steen, V. M., Andreassen, O. A., … Le Hellard, S. (2018). Recently evolved human-specific methylated regions are enriched in schizophrenia signals. BMC Evolutionary Biology, 18(63), 1–11. 10.1186/s12862-018-1177-2. 29747567 PMC5946405

[ref6] Bell, B. B., Abel, L. A., Li, W., Christian, J. C., & Yee, R. D. (1994). Concordance of smooth pursuit and saccadic measures in normal monozygotic twin pairs. Biological Psychiatry, 36(8), 522–526. 10.1016/0006-3223(94)90616-5. 7827215

[ref7] Braff, D. L., Freedman, R., Schork, N. J., & Gottesman, I. I. (2007). Deconstructing schizophrenia: An overview of the use of endophenotypes in order to understand a complex disorder. Schizophrenia Bulletin, 33(1), 21–32. 10.1093/schbul/sbl049. 17088422 PMC2632293

[ref8] Cain, S. M., & Snutch, T. P. (2011). Voltage-gated calcium channels and disease. BioFactors, 37(3), 197–205. 10.1002/biof.158. 21698699

[ref9] Calkins, M. E., Iacono, W. G., & Curtis, C. E. (2003). Smooth pursuit and antisaccade performance evidence trait stability in schizophrenia patients and their relatives. International Journal of Psychophysiology, 49(2), 139–146. 10.1016/S0167-8760(03)00101-6. 12919716

[ref10] Calkins, M. E., Iacono, W. G., & Ones, D. S. (2008). Eye movement dysfunction in first-degree relatives of patients with schizophrenia: A meta-analytic evaluation of candidate endophenotypes. Brain and Cognition, 68(3), 436–461. 10.1016/j.bandc.2008.09.001. 18930572 PMC4654966

[ref11] Campion, D., Thibaut, F., Denise, P., Courtin, P., Pottier, M., & Levillain, D. (1992). SPEM impairment in drug-naive schizophrenic patients: Evidence for a trait marker. Biological Psychiatry, 32(10), 891–902. 10.1016/0006-3223(92)90178-3. 1361365

[ref12] Chakravarti, A., & Little, P. (2003). Nature, nurture and human disease. Nature, 421(6921), 412–414. 10.1038/nature01401. 12540911

[ref13] Chasioti, D., Yan, J., Nho, K., & Saykin, A. J. (2019). Progress in polygenic composite scores in Alzheimer's and other complex diseases. Trends in Genetics, 35(5), 371–382. 10.1016/j.tig.2019.02.005. 30922659 PMC6475476

[ref14] Clementz, B. A., McDowell, J. E., & Zisook, S. (1994). Saccadic system functioning among schizophrenia patients and their first-degree biological relatives. Journal of Abnormal Psychology, 103(2), 277–287. 10.1037/0021-843X.103.2.277. 8040497

[ref15] Coors, A., Merten, N., Ward, D. D., Schmid, M., Breteler, M. M. B., & Ettinger, U. (2021). Strong age but weak sex effects in eye movement performance in the general adult population: Evidence from the Rhineland Study. Vision Research, 178, 124–133. 10.1016/j.visres.2020.10.004. 33387946

[ref16] Crevits, L., De Clerck, M., & Van Maele, G. (2000). Reliability of saccades. Neuro-Ophthalmology, 24(2), 319–325. 10.1076/noph.24.2.319.7159.

[ref17] Curtis, C. E., Calkins, M. E., Grove, W. M., Feil, K. J., & Iacono, W. G. (2001). Saccadic disinhibition in patients with acute and remitted schizophrenia and their first-degree biological relatives. American Journal of Psychiatry, 158(1), 100–106. 10.1176/appi.ajp.158.1.100. 11136640

[ref18] Damilou, A., Apostolakis, S., Thrapsanioti, E., Theleritis, C., & Smyrnis, N. (2016). Shared and distinct oculomotor function deficits in schizophrenia and obsessive compulsive disorder. Psychophysiology, 53(6), 796–805. 10.1111/psyp.12630. 26914941

[ref19] Diefendorf, A. R., & Dodge, R. (1908). An experimental study of the ocular reactions of the insane from photographic records. Brain, 31(3), 451–489. 10.1093/brain/31.3.451.

[ref20] Ettinger, U., Aichert, D. S., Wöstmann, N., Dehning, S., Riedel, M., & Kumari, V. (2018). Response inhibition and interference control: Effects of schizophrenia, genetic risk, and schizotypy. Journal of Neuropsychology, 12(3), 484–510. 10.1111/jnp.12126. 28485076

[ref21] Ettinger, U., Kumari, V., Crawford, T. J., Corr, P. J., Das, M., Zachariah, E., … Sharma, T. (2004). Smooth pursuit and antisaccade eye movements in siblings discordant for schizophrenia. Journal of Psychiatric Research, 38(2), 177–184. 10.1016/s0022-3956(03)00105-5. 14757332

[ref22] Ettinger, U., Kumari, V., Crawford, T. J., Davis, R. E., Sharma, T., & Corr, P. J. (2003). Reliability of smooth pursuit, fixation, and saccadic eye movements. Psychophysiology, 40(4), 620–628. 10.1111/1469-8986.00063. 14570169

[ref23] Ettinger, U., Picchioni, M., Hall, M. H., Schulze, K., Toulopoulou, T., Landau, S., … Murray, R. M. (2006). Antisaccade performance in monozygotic twins discordant for schizophrenia: The Maudsley twin study. American Journal of Psychiatry, 163(3), 543–545. 10.1176/appi.ajp.163.3.543. 16513882

[ref24] Flechtner, K.-M., Steinacher, B., Sauer, R., & Mackert, A. (2002). Smooth pursuit eye movements of patients with schizophrenia and affective disorder during clinical treatment. European Archives of Psychiatry and Clinical Neuroscience, 252(2), 49–53. 10.1007/s004060200011. 12111336

[ref25] Fox, J., & Weisberg, S. (2019). An {R} companion to applied regression (2nd ed.). Thousands Oaks, CA: SAGE. http://socserv.socsci.mcmaster.ca/jfox/Books/Companion.

[ref26] Fukushima, J., Fukushima, K., Chiba, T., Tanaka, S., Yamashita, I., & Kato, M. (1988). Disturbances of voluntary control of saccadic eye movements in schizophrenic patients. Biological Psychiatry, 23, 670–677. 10.1016/0006-3223(90)90060-F. 3370264

[ref27] Fukushima, J., Fukushima, K., Morita, N., & Yamashita, I. (1990a). Further analysis of the control of voluntary saccadic eye movements in schizophrenic patients. Biological Psychiatry, 28(11), 943–958. 10.1016/0006-3223(90)90060-F. 2275952

[ref28] Fukushima, J., Morita, N., Fukushima, K., Chiba, T., Tanaka, S., & Yamashita, I. (1990b). Voluntary control of saccadic eye movements in patients with schizophrenic and affective disorders. Journal of Psychiatric Research, 24(1), 9–24. 10.1016/0022-3956(90)90021-H. 2366215

[ref29] Gatt, J. M., Burton, K. L. O., Williams, L. M., & Schofield, P. R. (2015). Specific and common genes implicated across major mental disorders: A review of meta-analysis studies. Journal of Psychiatric Research, 60, 1–13. 10.1016/j.jpsychires.2014.09.014. 25287955

[ref30] Gooding, D. C., Mohapatra, L., & Shea, H. B. (2004). Temporal stability of saccadic task performance in schizophrenia and bipolar patients. Psychological Medicine, 34(5), 921–932. 10.1017/S003329170300165X. 15500312

[ref31] Gottesman, I. I., & Gould, T. D. (2003). The endophenotype concept in psychiatry: Etymology and strategic intentions. American Journal of Psychiatry, 160(4), 636–645. 10.1176/appi.ajp.160.4.636. 12668349

[ref32] Greenwood, T. A., Braff, D. L., Light, G. A., Cadenhead, K. S., Calkins, M. E., Dobie, D. J., … Schork, N. J. (2007). Initial heritability analyses of endophenotypic measures for schizophrenia. Archives of General Psychiatry, 64(11), 1242–1250. 10.1001/archpsyc.64.11.1242. 17984393 PMC10588564

[ref33] Greenwood, T. A., Light, G. A., Swerdlow, N. R., Radant, A. D., & Braff, D. L. (2012). Association analysis of 94 candidate genes and schizophrenia-related endophenotypes. PLoS ONE, 7(1), e29630. 10.1371/journal.pone.0029630. 22253750 PMC3258248

[ref34] Hallett, P. E. (1978). Primary and secondary saccades to goals defined by instructions. Vision Research, 18(10), 1279–1296. 10.1016/0042-6989(78)90218-3. 726270

[ref35] Halvorsen, M., Huh, R., Oskolkov, N., Wen, J., Netotea, S., Giusti-Rodriguez, P., … Szatkiewicz, J. P. (2020). Increased burden of ultra-rare structural variants localizing to boundaries of topologically associated domains in schizophrenia. Nature Communications, 11(1842), 1–13. 10.1038/s41467-020-15707-w. PMC7160146 32296054

[ref36] Haraldsson, H. M., Ettinger, U., Magnusdottir, B. B., Sigmundsson, T., Sigurdsson, E., Ingason, A., & Petursson, H. (2009). COMT val158met genotype and smooth pursuit eye movements in schizophrenia. Psychiatry Research, 169(2), 173–175. 10.1016/j.psychres.2008.10.003. 19647329

[ref37] Haraldsson, H. M., Ettinger, U., Magnusdottir, B. B., Sigmundsson, T., Sigurdsson, E., Ingason, A., & Petursson, H. (2010). Catechol-o-methyltransferase val158 met polymorphism and antisaccade eye movements in schizophrenia. Schizophrenia Bulletin, 36(1), 157–164. 10.1093/schbul/sbn064. 18562342 PMC2800134

[ref38] Harrell Jr F., & Dupont, C. (2020). *Hmisc: Harrell Miscellaneous*. *R package version* 4.3–1. https://cran.r-project.org/package=Hmisc.

[ref39] Herweg, N. A., Weber, B., Kasparbauer, A., Meyhöfer, I., Steffens, M., Smyrnis, N., & Ettinger, U. (2014). Functional magnetic resonance imaging of sensorimotor transformations in saccades and antisaccades. NeuroImage, 102(2), 848–860. 10.1016/j.neuroimage.2014.08.033. 25173413

[ref40] Holzman, P. S. (2000). Eye movements and the search for the essence of schizophrenia. Brain Research Reviews, 31(2–3), 350–356. 10.1016/S0165-0173(99)00051-X. 10719162

[ref41] Holzman, P. S., Proctor, L. R., & Hughes, D. W. (1973). Eye-tracking patterns in schizophrenia. Science (New York, N.Y.), 181(4095), 179–181. 4711736 10.1126/science.181.4095.179

[ref42] Hong, L. E., Mitchell, B. D., Avila, M. T., Adami, H., Mcmahon, R. P., & Thaker, G. K. (2006). Familial aggregation of eye-tracking endophenotypes in families of schizophrenic patients. Archives of General Psychiatry, 63(3), 259–264. 10.1001/archpsyc.63.3.259. 16520430

[ref43] Howie, B. N., Donnelly, P., & Marchini, J. (2009). A flexible and accurate genotype imputation method for the next generation of genome-wide association studies. PLoS Genetics, 5(6), e1000529. 10.1371/journal.pgen.1000529. 19543373 PMC2689936

[ref44] Hutton, S. B. (2008). Cognitive control of saccadic eye movements. Brain and Cognition, 68(3), 327–340. 10.1016/j.bandc.2008.08.021. 19028265

[ref45] Iacobucci, D., Schneider, M. J., Popovich, D. L., & Bakamitsos, G. A. (2016). Mean centering helps alleviate ‘micro’ but not ‘macro’ multicollinearity. Behavior Research Methods, 48(4), 1308–1317. 10.3758/s13428-015-0624-x. 26148824

[ref1] International Schizophrenia Consortium (2009). Common polygenic variation contributes to risk of schizophrenia that overlaps with bipolar disorder. Nature, 460(7256), 748–752. 10.1038/nature08185. 19571811 PMC3912837

[ref46] Jonas, K. G., Lencz, T., Li, K., Malhotra, A. K., Perlman, G., Fochtmann, L. J., … Kotov, R. (2019). Schizophrenia polygenic risk score and 20-year course of illness in psychotic disorders. Translational Psychiatry, 9, 1–8. 10.1038/s41398-019-0612-5. 31727878 PMC6856168

[ref47] Karoumi, B., Saoud, M., D'Amato, T., Rosenfeld, F., Denise, P., Gutknecht, C., … Rochet, T. (2001). Poor performance in smooth pursuit and antisaccadic eye-movement tasks in healthy siblings of patients with schizophrenia. Psychiatry Research, 101(3), 209–219. 10.1016/S0165-1781(01)00227-X. 11311924

[ref48] Katsanis, J., Taylor, J., Iacono, W. G., & Hammer, M. A. (2000). Heritability of different measures of smooth pursuit eye tracking dysfunction: A study of normal twins. Psychophysiology, 37(6), 724–730. 10.1111/1469-8986.3760724. 11117452

[ref49] Kattoulas, E., Stefanis, N. C., Avramopoulos, D., Stefanis, C. N., Evdokimidis, I., & Smyrnis, N. (2012). Schizophrenia-related RGS4 gene variations specifically disrupt prefrontal control of saccadic eye movements. Psychological Medicine, 42(4), 757–767. 10.1017/S003329171100167X. 21910931

[ref50] Kaufman, L. D., Pratt, J., Levine, B., & Black, S. E. (2010). Antisaccades: A probe into the dorsolateral prefrontal cortex in Alzheimer's disease. A critical review. Journal of Alzheimer's Disease, 19, 781–793. 10.3233/JAD-2010-1275. 20157236

[ref51] Keedy, S. K., Ebens, C. L., Keshavan, M. S., & Sweeney, J. A. (2006). Functional magnetic resonance imaging studies of eye movements in first episode schizophrenia: Smooth pursuit, visually guided saccades and the oculomotor delayed response task. Psychiatry Research – Neuroimaging, 146(3), 199–211. 10.1016/j.pscychresns.2006.01.003. 16571373

[ref52] Levy, D. L., Sereno, A. B., Gooding, D. C., & O'Driscoll, G. A. (2010). Eye tracking dysfunction in schizophrenia: Characterization and pathophysiology. Current Topics in Behavioral Neurosciences, 4, 311–347. 10.1007/7854_2010_60. 21312405 PMC3212396

[ref53] Lichtenstein, P., Björk, C., Hultman, C. M., Scolnick, E., Sklar, P., & Sullivan, P. F. (2006). Recurrence risks for schizophrenia in a Swedish National Cohort. Psychological Medicine, 36(10), 1417–1425. 10.1017/S0033291706008385. 16863597

[ref54] Lichtenstein, P., Yip, B. H., Björk, C., Pawitan, Y., Cannon, T. D., Sullivan, P. F., … Hultman, C. M. (2009). Common genetic influences for schizophrenia and bipolar disorder: A population-based study of 2 million nuclear families. Lancet (London, England), 17(373), 9659. 10.1016/S0140-6736(09)60072-6. PMC3879718 19150704

[ref55] Light, G. A., Swerdlow, N. R., Rissling, A. J., Radant, A., Sugar, C. A., Sprock, J., … Braff, D. L. (2012). Characterization of neurophysiologic and neurocognitive biomarkers for use in genomic and clinical outcome studies of schizophrenia. PLoS ONE, 7(7), e39434. 10.1371/journal.pone.0039434. 22802938 PMC3389010

[ref56] Litman, R. E., Torrey, E. F., Hommer, D. W., Radant, A. R., Pickar, D., & Weinberger, D. R. (1997). A quantitative analysis of smooth pursuit eye tracking in monozygotic twins discordant for schizophrenia. Archives of General Psychiatry, 54(5), 417–426. 10.1001/archpsyc.1997.01830170035006. 9152095

[ref57] Macare, C., Meindl, T., Nenadic, I., Rujescu, D., & Ettinger, U. (2014). Preliminary findings on the heritability of the neural correlates of response inhibition. Biological Psychology, 103(1), 19–23. 10.1016/j.biopsycho.2014.07.017. 25101865

[ref58] Malone, S. M., & Iacono, W. G. (2002). Error rate on the antisaccade task: Heritability and developmental change in performance among preadolescent and late-adolescent female twin youth. Psychophysiology, 39(5), 664–673. 10.1017/S004857720201079X. 12236334

[ref59] Marees, A. T., de Kluiver, H., Stringer, S., Vorspan, F., Curis, E., Marie-Claire, C., & Derks, E. M. (2018). A tutorial on conducting genome-wide association studies: Quality control and statistical analysis. International Journal of Methods in Psychiatric Research, 27(2), 1–10. 10.1002/mpr.1608. PMC6001694 29484742

[ref60] Marshall, C. R., Howrigan, D. P., Merico, D., Bhooma, T., Wu, W., Greer, D. S., … Maile, M. S. (2017). Contribution of copy number variants to schizophrenia from a genome-wide study of 41321 subjects. Nature Genetics, 49(1), 27–35. 10.1038/ng.3725. 27869829 PMC5737772

[ref61] Mazhari, S., Price, G., Dragović, M., Waters, F. A., Clissa, P., & Jablensky, A. (2011). Revisiting the suitability of antisaccade performance as an endophenotype in schizophrenia. Brain and Cognition, 77(2), 223–230. 10.1016/j.bandc.2011.08.006. 21924537

[ref62] McDowell, J. E., Brown, G. G., Paulus, M., Martinez, A., Stewart, S. E., Dubowitz, D. J., & Braff, D. L. (2002). Neural correlates of refixation saccades and antisaccades in normal and schizophrenia subjects. Biological Psychiatry, 51(3), 216–223. 10.1016/S0006-3223(01)01204-5. 11839364

[ref63] McGrath, J., Saha, S., Chant, D., & Welham, J. (2008). Schizophrenia: A concise overview of incidence, prevalence, and mortality. Epidemiologic Reviews, 30(1), 67–76. 10.1093/epirev/mxn001. 18480098

[ref64] Moon, S. Y., Barton, J. J. S., Mikulski, S., Polli, F. E., Cain, M. S., Vangel, M., … Manoach, D. S. (2007). Where left becomes right: A magnetoencephalographic study of sensorimotor transformation for antisaccades. NeuroImage, 36(4), 1313–1323. 10.1016/j.neuroimage.2007.04.040. 17537647 PMC1995561

[ref65] Munoz, D. P., & Everling, S. (2004). Look away: The anti-saccade task and the voluntary control of eye movement. Nature Reviews Neuroscience, 5(3), 218–228. 10.1038/nrn1345. 14976521

[ref66] O'Driscoll, G. A., & Callahan, B. L. (2008). Smooth pursuit in schizophrenia: A meta-analytic review of research since 1993. Brain and Cognition, 68(3), 359–370. 10.1016/j.bandc.2008.08.023. 18845372

[ref67] Pangrsic, T., Singer, J. H., & Koschak, A. (2018). Voltage-gated calcium channels: Key players in sensory coding in the retina and the inner ear. Physiological Reviews, 98(4), 2063–2096. 10.1152/physrev.00030.2017. 30067155 PMC6170976

[ref68] Pardiñas, A. F., Holmans, P., Pocklington, A. J., Escott-Price, V., Ripke, S., Carrera, N., … Walters, J. T. R. (2018). Common schizophrenia alleles are enriched in mutation-intolerant genes and in regions under strong background selection. Nature Genetics, 50, 381–389. 10.1038/s41588-018-0059-2. 29483656 PMC5918692

[ref69] Price, A. L., Patterson, N. J., Plenge, R. M., Weinblatt, M. E., Shadick, N. A., & Reich, D. (2006). Principal components analysis corrects for stratification in genome-wide association studies. Nature Genetics, 38(8), 904–909. 10.1038/ng1847. 16862161

[ref70] Purcell, S. M., Neale, B., Todd-Brown, K., Thomas, L., Ferreira, M. A. R., Bender, D., … Sham, P. C. (2007). PLINK: A tool set for whole-genome association and population-based linkage analyses. American Journal of Human Genetics, 81(3), 559–575. 10.1086/519795. 17701901 PMC1950838

[ref71] Radant, A. D., Dobie, D. J., Calkins, M. E., Olincy, A., Braff, D. L., Cadenhead, K. S., … Tsuang, D. W. (2007). Successful multi-site measurement of antisaccade performance deficits in schizophrenia. Schizophrenia Research, 89(1–3), 320–329. 10.1016/j.schres.2006.08.010. 17023145

[ref72] Radant, A. D., Dobie, D. J., Calkins, M. E., Olincy, A., Braff, D. L., Cadenhead, K. S., … Tsuang, D. W. (2010). Antisaccade performance in schizophrenia patients, their first-degree biological relatives, and community comparison subjects: Data from the COGS study. Psychophysiology, 47(5), 846–856. 10.1111/j.1469-8986.2010.01004.x. 20374545 PMC4176871

[ref73] Raemaekers, M., Jansma, J. M., Cahn, W., Van der Geest, J., van der Linden, J. A., Kahn, R. S., & Ramsey, N. F. (2002). Neuronal substrate of the saccadic inhibition deficit in schizophrenia investigated with 3-dimensional event-related functional magnetic resonance imaging. Archives of General Psychiatry, 59(4), 313–320. 10.1001/archpsyc.59.4.313. 11926931

[ref74] Raemaekers, M., Ramsey, N. F., Vink, M., van den Heuvel, M. P., & Kahn, R. S. (2006). Brain activation during antisaccades in unaffected relatives of schizophrenic patients. Biological Psychiatry, 59(6), 530–535. 10.1016/j.biophych.2005.07.030. 16165103

[ref75] Rajji, T. K., Ismail, Z., & Mulsant, B. H. (2009). Age at onset and cognition in schizophrenia: Meta-analysis. British Journal of Psychiatry, 195(4), 286–293. 10.1192/bjp.bp.108.060723. 19794194

[ref76] Reilly, J. L., Frankovich, K., Hill, S., Gershon, E. S., Keefe, R. S. E., Keshavan, M. S., … Sweeney, J. A. (2014). Elevated antisaccade error rate as an intermediate phenotype for psychosis across diagnostic categories. Schizophrenia Bulletin, 40(5), 1011–1021. 10.1093/schbul/sbt132. 24080895 PMC4133662

[ref77] Reuter, B., Rakusan, L., & Kathmanna, N. (2005). Poor antisaccade performance in schizophrenia: An inhibition deficit? Psychiatry Research, 135(1), 1–10. 10.1016/j.psychres.2004.12.006. 15893384

[ref78] Ripke, S., Neale, B. M., Corvin, A., Walters, J. T. R., Farh, K. H., Holmans, P. A., … O'Donovan, M. C. (2014). Biological insights from 108 schizophrenia-associated genetic loci. Nature, 511(7510), 421–427. 10.1038/nature13595. 25056061 PMC4112379

[ref79] Rothman, K. J. (1990). No adjustments are needed for multiple comparisons. Epidemiology (Cambridge, Mass.), 1(1), 43–46. 10.1016/S0360-3199(98)00119-0. 2081237

[ref80] Rybakowski, J. K., Borkowska, A., Czerski, P. M., & Hauser, J. (2002). Eye movement disturbances in schizophrenia and a polymorphism of catechol-O-methyltransferase gene. Psychiatry Research, 113(1–2), 49–57. 10.1016/S0165-1781(02)00245-7. 12467945

[ref81] Schmid-Burgk, W. (1984). Sakkadische Augenbewegungen und endogene Psychosen. Fortschritte Der Neurologie Psychiatrie, 52(2), 67–71. 10.1055/s-2007-1002003. 6714915

[ref82] Schreiber, H., Rothmeier, J., Becker, W., Jürgens, R., Born, J., Stolz-Born, G., … Kornhuber, H. H. (1995). Comparative assessment of saccadic eye movements, psychomotor and cognitive performance in schizophrenics, their first-degree relatives and control subjects. Acta Psychiatrica Scandinavica, 91, 195–201. 7625195 10.1111/j.1600-0447.1995.tb09766.x

[ref83] Sereno, A. B., & Holzman, P. S. (1995). Antisaccades and smooth pursuit eye movements in schizophrenia. Biological Psychiatry, 37(6), 394–401. 10.1016/0006-3223(94)00127-O. 7772648

[ref84] Siegel, J. J., Chitwood, R. A., Ding, J. M., Payne, C., Taylor, W., Gray, R., … Johnston, D. (2017). Prefrontal cortex dysfunction in fragile X mice depends on the continued absence of fragile X mental retardation protein in the adult brain. Journal of Neuroscience, 37(31), 7305–7317. 10.1523/JNEUROSCI.0571-17.2017. 28652410 PMC6596701

[ref85] Smeland, O. B., Frei, O., Dale, A. M., & Andreassen, O. A. (2020). The polygenic architecture of schizophrenia - rethinking pathogenesis and nosology. Nature Reviews Neurology, 16, 366–379. 10.1038/s41582-020-0364-0. 32528109

[ref86] Srinivasan, S., Bettella, F., Mattingsdal, M., Wang, Y., Witoelar, A., Schork, A. J., … Andreassen, O. A. (2016). Genetic markers of human evolution are enriched in schizophrenia. Biological Psychiatry, 80(4), 284–292. 10.1016/j.biopsych.2015.10.009. 26681495 PMC5397584

[ref87] Streiner, D. L., & Norman, G. R. (2011). Correction for multiple testing: Is there a resolution? Chest, 140(1), 16–18. 10.1378/chest.11-0523. 21729890

[ref88] Sullivan, P. F., Kendler, K. S., & Neale, M. C. (2003). Schizophrenia as a complex trait: Evidence from a meta-analysis of twin studies. Archives of General Psychiatry, 60(12), 1187–1192. 10.1001/archpsyc.60.12.1187. 14662550

[ref89] Sweeney, J. A., Luna, B., Haas, G. L., Keshavan, M. S., Mann, J. J., & Thase, M. E. (1999). Pursuit tracking impairments in schizophrenia and mood disorders: Step-ramp studies with unmedicated patients. Biological Psychiatry, 46(5), 671–680. 10.1016/S0006-3223(99)00132-8. 10472419

[ref90] Sweeney, J. A., Rosanao, C., Berman, R. A., & Luna, B. (2001). Inhibitory control of attention declines more than working memory during normal aging. Neurobiology of Aging, 22(1), 39–47. 10.1016/S0197-4580(00)00175-5. 11164275

[ref91] Thaker, G. K., Wonodi, I., Avila, M. T., Hong, L. E., & Stine, O. C. (2004). Catechol o-methyltransferase polymorphism and eye tracking in schizophrenia: A preliminary report. American Journal of Psychiatry, 161(12), 2320–2322. 10.1176/appi.ajp.161.12.2320. 15569909

[ref2] The Brainstorm Consortium (2018). Analysis of shared heritability in common disorders of the brain. Science (New York, N.Y.), 360(6395), eaap8757. 10.1126/science.aap8757. 29930110 PMC6097237

[ref92] Toulopoulou, T., Zhang, X., Cherny, S., Dickinson, D., Berman, K. F., Straub, R. E., … Weinberger, D. R. (2019). Polygenic risk score increases schizophrenia liability through cognition-relevant pathways. Brain, 142(2), 471–485. 10.1093/brain/awy279. 30535067 PMC6359897

[ref93] Verma, S. S., de Andrade, M., Tromp, G., Kuivaniemi, H., Pugh, E., Namjou-Khales, B., … Ritchie, M. D. (2014). Imputation and quality control steps for combining multiple genome-wide datasets. Frontiers in Genetics, 5, 370. 10.3389/fgene.2014.00370. 25566314 PMC4263197

[ref94] Wand, H., Lambert, S. A., Tamburro, C., Iacocca, M. A., O'Sullivan, J. W., Sillari, C., … Antoniou, A. C. (2021). Improving reporting standards for polygenic scores in risk prediction studies. Nature, 591, 211–219. 10.1038/s41586-021-03243-6. 33692554 PMC8609771

[ref95] Zhang, J. P., Robinson, D., Yu, J., Gallego, J., Fleischhacker, W. W., Kahn, R. S., … Lencz, T. (2019). Schizophrenia polygenic risk score as a predictor of antipsychotic efficacy in first-episode psychosis. American Journal of Psychiatry, 176(1), 21–28. 10.1176/appi.ajp.2018.17121363. 30392411 PMC6461047

