# Supplementary material for: Polygenic risk scores for schizophrenia are associated with oculomotor endophenotypes
Source: Psychol Med. 2021 Aug 20;53(4):1611–9. doi: 10.1017/S0033291721003251 (PMC10009390; doi:10.1017/S0033291721003251)
Supplement: Supplementary file 1 [file S0033291721003251sup001.pdf]

Polygenic Risk Scores for Schizophrenia Are Associated with Oculomotor Endophenotypes – Supplementary Material

Annabell Coors[a], Mohammed-Aslam Imtiaz[a], Meta M. Boenniger[a], N. Ahmad Aziz[a,b], Monique M.B. Breteler[a,c,#,+] & Ulrich Ettinger[d,#]

[a]*Population Health Sciences, German Center for Neurodegenerative Diseases (DZNE), Bonn, Germany*

[b]*Department of Neurology, Faculty of Medicine, University of Bonn, Germany*

[c]*Institute for Medical Biometry, Informatics and Epidemiology (IMBIE), Faculty of Medicine, University of Bonn, Germany*

[d]*Department of Psychology, University of Bonn, Germany*

[#] These authors contributed equally to this paper

[+]Corresponding author

Address correspondence to:

Monique M. B. Breteler, DZNE, Venusberg-Campus 1, Gebäude 99, 53127 Bonn, Germany.

Email: monique.breteler@dzne.de; Phone: +49 228 43302 929

Table A.1 Associations between Polygenic Risk Scores (PRS) for Schizophrenia at Different p-value Thresholds for SNP Inclusion and Eye Movement Outcomes

**Eye Movement Outcome**	**p-value Threshold for SNP Inclusion**	**b (95%-CI) for PRS**	**p-value**	**R^2 (%)**
Antisaccade error rate (%)	$5*10^{-8}$	0.743 (-0.639, 2.125)	0.292	/
Antisaccade error rate (%)	0.1	0.864 (-0.069, 1.796)	0.069	/
Log of antisaccade latency (log ms)	$5*10^{-8}$	0.003 (-0.001, 0.007)	0.153	/
Log of antisaccade latency (log ms)	0.1	0.002 (-0.001, 0.005)	0.154	/
Antisaccade amplitude gain (%)	$5*10^{-8}$	-1.667 (-3.457, 0.123)	0.068	/
Antisaccade amplitude gain (%)	0.1	-1.088 (-2.299, 0.124)	0.078	/
Smooth pursuit velocity gain (%)	$5*10^{-8}$	-0.014 (-0.884, 0.857)	0.976	/
Smooth pursuit velocity gain (%)	0.1	**0.687 (0.100, 1.273)**	**0.022**	**0.14**
Saccade frequency during smooth pursuit (N/s)	$5*10^{-8}$	0.004 (-0.028, 0.037)	0.790	/
Saccade frequency during smooth pursuit (N/s)	0.1	-0.001 (-0.023, 0.020)	0.902	/
Prosaccade amplitude gain (%)	$5*10^{-8}$	-0.095 (-0.497, 0.307)	0.643	/
Prosaccade amplitude gain (%)	0.1	-0.079 (-0.350, 0.191)	0.564	/
Log of prosaccade latency (log ms)	$5*10^{-8}$	0.000 (-0.003, 0.003)	0.979	/
Log of prosaccade latency (log ms)	0.1	-0.002 (-0.004, 0.000)	0.126	/

Note. The table displays the change in performance per one standard deviation increase in PRS for schizophrenia for different eye movement outcomes. b=unstandardized regression coefficient, 95%-CI=95%-confidence interval. Unstandardized regression coefficients were obtained from the following multivariable linear regression model: Eye movement outcome ~ b_0 + PRS* b_1 + age + age^2 + sex + population stratification + residual error. R^2 refers to the variance explained in eye movement performance by PRS in percent.
